# The cognitive underpinnings of minority overestimation

**DOI:** 10.1073/pnas.2527709123

**Published:** 2026-02-23

**Authors:** Rasha Kardosh, Asael Y. Sklar

**Affiliations:** ^a^Department of Psychology, New York University, New York, NY 10003; ^b^Arison School of Business, Reichman University, Herzliya 4634865, Israel

People consistently overestimate the size of minority groups, and this misperception correlates with adversarial attitudes, including anti-immigrant sentiment and support for discriminatory policies (1, 2). Guay, Marghetis, Wong, and Landy (henceforth, GMWL) attribute these overestimations to uncertainty-based rescaling (UBR). In this account, when people provide explicit survey estimates, uncertainty pushes judgments toward prior expectations about group sizes, therefore producing systematic overestimation of small groups (3).

In work previously published in this journal, we reported experimental findings indicating that minority overestimation is not merely a product of rescaling during survey response; it also reflects how people perceive social settings. Cognition is tuned to attend to (4), remember (5), and thus overweight rare events (6, 7). In social perception, these processes apply to minorities who are, by definition, relatively rare. Minorities thus become salient in perception, memory, and visual awareness, leading people to overestimate their true prevalence (8). This account diverges from GMWL’s UBR model on a key point: Overestimation is not only the result of processes that produce explicit survey estimates but also of processes that shape everyday experience.

While both mechanisms are domain-general and may produce similar mathematical predictions for survey responses, experimental manipulations provide key empirical contrasts. In our studies (8), we systematically varied the proportions of Black and White faces shown to participants (8). These experiments allowed separating the mechanisms in two ways.

## Overestimation beyond 50%

**Fig. 1.**
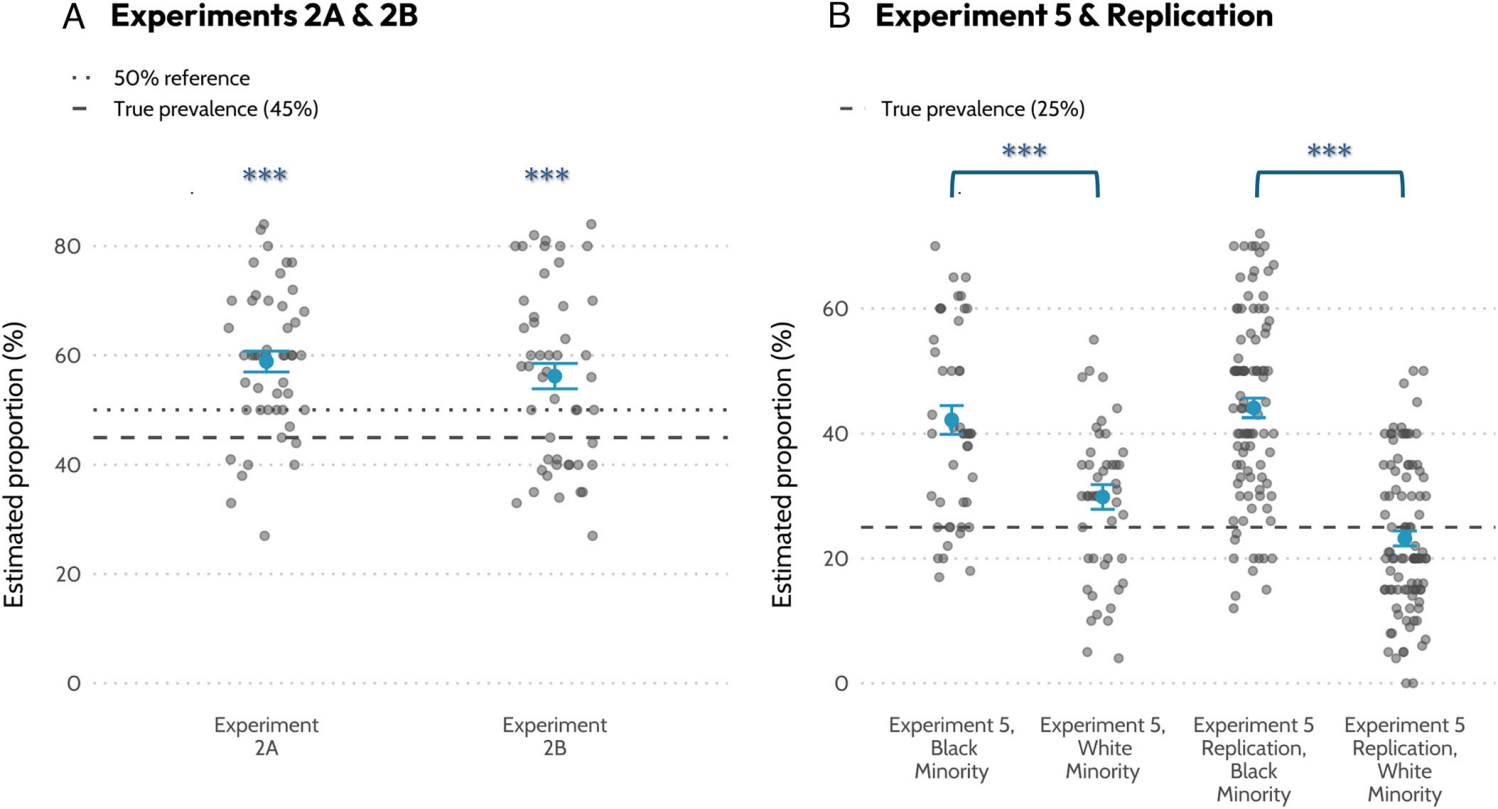
Overestimation of minority prevalence across experiments. (*A*) In Experiments 2A and 2B (8), participants judged the prevalence of Black-American faces when the true proportion was set to 45% (dashed line). Estimates significantly exceeded both the true proportion, and the 50% reference line (dotted line). Blue circles indicate condition means, with error bars representing ±1 SEM. Asterisks indicate significant overestimation relative to 50% (***P < 0.001). (*B*) In Experiment 5 and its preregistered replication (8, 9), under the same prevalence (25%), Black minorities were overestimated substantially more than White minorities. The dashed line indicates the true prevalence of both minorities (25%). Blue circles indicate condition means, with error bars representing ±1 SEM. Brackets denote significant differences between Black and White Minority conditions (***P < 0.001).

In experiments where minority prevalence was set at 45%, participants’ estimates were not only significantly higher than 45% but also significantly exceeded 50% (8, Experiments 2A,2B; Fig. 1). Within GMWL’s model, where minority priors are bounded between 0% and 50%, rescaling toward such priors cannot generate estimates significantly exceeding 50%. By contrast, a salience-based mechanism predicts minority groups may retain salience even when numerically prevalent.

## Artificially Minoritized Groups

In experiments where White faces were made the artificial minority (25% of displayed faces), participants overestimated this prevalence to a lesser extent than when Black faces were set to the same prevalence (8, Experiment 5; 9; Fig. 1). This asymmetry conflicts with UBR predictions, unless one assumes, implausibly, that priors are higher for minority than majority prevalence. A salience account, where attention amplifies minority presence, naturally produces this pattern.

Of course, both mechanisms likely contribute to minority overestimation. Even when salience is minimal (e.g., people living east of the Mississippi), UBR may produce overestimation. However, in estimates relating to experience and memory, understanding that both mechanisms play a role matters for interpretation and communication. GMWL urge researchers, journalists, and commentators to adopt psychologically realistic accounts of minority overestimation. This requires recognizing that cognition shapes impressions of social environments, not only survey responses. Although survey-based data may not provide clear connections between minority overestimation and public attitudes at the individual-differences level, experimental data establish a causal link at the group level: In our work, participants informed of true minority prevalence were more supportive of diversity-promoting policies than those relying on biased impressions (8, Experiment 6). Thus, accurate communication of minority prevalence can temper public opinion and foster support for inclusive policies.
